# Post-traumatic headache in patients with minimal traumatic intracranial hemorrhage after traumatic brain injury: a retrospective matched case-control study

**DOI:** 10.1186/s10194-017-0774-6

**Published:** 2017-06-26

**Authors:** Chang-Ki Hong, Yu Shik Shim, Sook Young Sim, Jin-Yang Joo, Min A Kwon, Yong Bae Kim, Joonho Chung

**Affiliations:** 1 Department of Neurosurgery, Gangnam Severance Hospital, Yonsei University College of Medicine, 211, Eonjuro, Gangnam-gu, Seoul, 06273 Republic of Korea; 2 Severance Institute for Vascular and Metabolic Research, Yonsei University, Seoul, Republic of Korea; 3 Department of Neurosurgery, Inha University School of Medicine and Hospital, Incheon, Republic of Korea; 4 Department of Neurosurgery, Inje University Seoul Paik Hospital, Seoul, Republic of Korea; 5 Department of Neurological Surgery, Rush University Medical Center, 1725 W. Harrison St., Professional Building Suite 855, Chicago, IL 60612 USA

**Keywords:** Head trauma, Post-traumatic headache, Traumatic brain injury, Traumatic intracranial hemorrhage

## Abstract

**Background:**

No evidence is available on the risks of neurologically asymptomatic minimal traumatic intracranial hemorrhage (mTIH) in patients with traumatic brain injury (TBI) for post-traumatic headache (PTH). The purpose of this study was to investigate whether mTIH in patients with TBI was associated with PTH and to evaluate its risk factors.

**Methods:**

Between September 2009 and December 2014, 1484 patients with TBI were treated at our institution, 57 of whom had mTIH after TBI and were include in this study. We performed propensity score matching to establish a control group among the 823 patients with TBI treated during the same period. Patients with TBI rated their headaches prospectively using a numeric rating scale (NRS). We compared NRS scores between mTIH group (*n* = 57) and non-mTIH group (*n* = 57) and evaluated risk factors of moderate-to-severe PTH (NRS ≥ 4) at the 12-month follow-up.

**Results:**

Moderate-to-severe PTH was reported by 21.9% of patients (29.8% in mTIH group and 14.0% in non-mTIH group B, *p* = 0.012) at the 12-month follow-up. The mean NRS was higher in mTIH group than in non-mTIH group throughout the follow-up period (95% confidence interval [CI], 0.11 to 1.14; *p* < 0.05, ANCOVA). Logistic regression analysis showed that post-traumatic seizure (odds ratio, 1.520; 95% CI, 1.128–6.785; *p* = 0.047) and mTIH (odds ratio, 2.194; 95% CI, 1.285–8.475; *p* = 0.039) were independently associated with moderate-to-severe PTH at the 12-month follow-up.

**Conclusions:**

Moderate-to-severe PTH can be expected after TBI in patients with mTIH and post-traumatic seizure. PTH occurs more frequently in patients with mTIH than in those without mTIH.

## Background

Patients with traumatic brain injury (TBI) can have only a brief change in mental status, such as confusion, disorientation, loss of memory, or loss of consciousness (LOC) for less than 30 min, without serious permanent neurological deficits even if they have abnormal radiological findings. Previously, studies that addressed patients with neurologically asymptomatic minimal traumatic intracranial hemorrhage (mTIH) received little attention as the clinical course of most patients with mTIH is good, without morbidity or mortality. However, they frequently experience post-traumatic headache (PTH) that may affect the quality of life [1]. Little is known about PTH in patients with mTIH as, ultimately, achieving a good clinical outcome (to minimize morbidity and mortality) in patients with severe intracranial findings is of higher importance than PTH.

PTH refers to a headache that develops within 1 week after head trauma. During the first 3 months from onset, PTH is considered acute. Persistent PTH was described as headache of greater than 3 months’ duration caused by traumatic injury to the head. The term persistent has been adopted in place of chronic [2]. Although most PTHs resolve within 6 to 12 months after injury, approximately 18–33% of PTHs persist beyond 12 months [3]. PTH is known to be related to other problems, such as depression, cognitive dysfunction, or insomnia [4]. However, risk factors for development of PTH in patients with mTIH after TBI have not been determined. The incidence of PTH and its impact may be different in patients with mTIH than in those with without mTIH. Therefore, the purpose of this matched case-control study was therefore to investigate whether mTIH in patients with TBI was associated with PTH and to evaluate its risk factors.

## Methods

This study, a retrospective case-control study from a prospectively collected database, was approved by our institutional review board, and informed consent was waived. Between September 2009 and December 2014, 1484 patients with TBI rated their headaches prospectively using a numeric rating scale (NRS). Among these patients, 57 (mTIH group) were included in the present study as they met the following criteria: (1) presence of headache due to head trauma; (2) presence of traumatic intracranial hemorrhage on radiographic images (computed tomography [CT] or magnetic resonance image [MRI]); (3) age ≥ 15 years; (4) initial Glasgow Coma Scale (GCS) ≥ 13; and (5) transient LOC ≤ 30 min. Of the 1427 remaining patients with TBI in the database, 604 were excluded for the following reasons: (1) initial GCS < 13 (*n* = 235); (2) initial or follow-up modified Rankin Scale (mRS) ≥ 3 (*n* = 45); (3) history of neurosurgery (craniotomy due to any cause) (*n* = 29); (4) TBI due to any cerebrovascular disease (*n* = 81); (5) history of previously diagnosed intracranial disease (*n* = 34); (5) post-traumatic amnesia >24 h (*n* = 22); (6) delayed hemorrhage (>24 h) (*n* = 22); (7) combined spinal cord injury (*n* = 74); (8) incomplete NRS score data (*n* = 14) and (9) loss to follow-up (*n* = 48). Among the remaining 823 patients, we performed propensity score matching to establish a proper control group (*n* = 57, non-mTIH group). All of them have experienced headache due to head trauma. Our inclusion/exclusion criteria have contained the definition of mild TBI since mild TBI, in general, is defined as TBI resulting in GCS ≥ 13, LOC ≤ 30 min, and post-traumatic amnesia ≤24 h [5, 6]. According to the GCS scoring system, moderate TBI is defined as a GCS of 9 to 12 and severe TBI is defined as a GCS of 3 to 8.p-h.

Three independent investigators blinded to the data retrospectively reviewed the clinical and radiographic data of the included patients using their medical records. Variables evaluated and compared between groups were age, gender, initial GCS score, cause of TBI, mechanism of TBI, type of mTIH, initial symptoms and signs (LOC, vomiting, tinnitus, visual symptoms, confusion, post-traumatic amnesia, and post-traumatic seizure), previously physician-diagnosed headache (treated by medications), and use of antiplatelet agent and/or anticoagulant. Anticonvulsant agents including topiramate or valproate have been given to all patients who experienced post-traumatic seizures.

Intensity of headache was assessed with the NRS, an 11-point numeric scale for rating pain intensity. The quantitative scale ranges from 0 to 10, with 0 meaning “no headache at all” and 10 meaning “the worst possible headache.” This 11-point scale has been previously applied to headache assessment [7–9]. We defined NRS scores of 1 to 3, 4 to 6, and 7 to 10 as mild, moderate, and severe headache, respectively. Mild headache was described as nagging, annoying, or interfering little with active daily living. Moderate headache was described as interfering significantly with active daily living. Severe headache was described as disabling or interfering to the point that patients were unable to perform active daily activities. Thus, we defined an NRS score ≥ 4 as significant. All included patients visited the emergency department or the outpatient office within 48 h of onset. NRS score was determined every 8 h (three times per day) during a patient’s hospitalization and twice during each outpatient visit. Analgesics were prescribed to patients with headache. Patient NRS scores were collected and averaged for the on initial visit and at 1-week, 1-month, 3-month, 6-month, and 12-month follow-ups. We compared the NRS and its changes between groups and evaluated risk factors of moderate-to-severe PTH (NRS ≥ 4) at the 12-month follow-up.

**Fig. 1:**
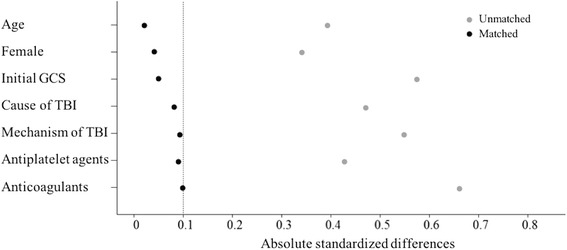
Absolute standardized difference (ASD) before and after propensity score matching. ASD < 0.10 implies good balance between the two groups. GCS, Glasgow Coma Scale; TBI, traumatic brain injury

Propensity score matching was performed using multiple logistic regression to match patients who had mTIH with those that did not have mTIH with respect to age, gender, initial GCS score, and cause of TBI. After patient matching by estimated propensity scores via a conditional logistic regression method, multiple logistic regression analysis was performed. Using the logit estimated from the log odds of the propensity score of each patient, we matched the selected cases with controls who had the nearest estimated logit value by 1:1 matching. Balance between mTIH group and non-mTIH group for each variable was evaluated by propensity score distributions and absolute standardized differences (ASD) before and after matching were calculated (Fig. 1). An ASD < 0.10 implies good balance between the two groups. A complete set of baseline data is essential to develop a propensity model. Thus, we replaced missing data values with the mean for that variable instead of excluding patients with missing data.

### Statistical analysis

All statistical analyses were consulted with a biostatistician and were performed using R language ver. 3.01 (R Foundation for Statistical Computing, Vienna, Austria). Student’s t tests were used for numeric variables. Chi-square tests were used for nominal variables. An ANCOVA with a general linear model, with group and follow-up time point as fixed factors, was used to compare the average NRS scores at each follow-up period between the two groups. Logistic regression analysis was performed to determine the independent risk factors of moderate-to-severe PTH at the 12-month follow-up. Multiple logistic regression analysis was performed on variables with an unadjusted effect and a *p*-value <0.10 on simple logistic regression analysis. Statistical significance was defined by a *p*-value <0.05 with a 95% confidence interval.

## Results

The clinical characteristics of propensity score-matched subjects in both groups are presented in Table 1. Mean age was 34.7 ± 11.2 years in mTIH group and 34.4 ± 11.9 years in non-mTIH group (*p* = 0.916). There were 25 (43.9%) females in mTIH group and 26 (45.6%) in non-mTIH group (*p* = 0.864), while 43.9% of patients in mTIH group and 47.4% in non-mTIH group had an initial GCS score of 15 (*p* = 0.838). Motor vehicle accident (59.6% in mTIH group and 57.9% in non-mTIH group) was the most frequent cause of TBI (*p* = 0.782). Head striking an object (68.4% in mTIH group and 63.2% in non-mTIH group) was the most frequent mechanism of TBI, followed by head being struck (19.3% in mTIH group and 21.1% in non-mTIH group) and whiplash (12.3% in mTIH group and 15.8% in non-mTIH group) (*p* = 0.705). In mTIH group, subarachnoid hemorrhage was the most frequent mTIH (68.4%), followed by subdural hemorrhage (49.1%), epidural hemorrhage (29.8%), intraventricular hemorrhage (7.0%), and contusional hemorrhage (3.5%).

**Table 1:** Clinical characteristics of propensity score-matched subjects

	mTIH (*n* = 57)	non-mTIH (*n* = 57)	*p* value
Age (mean ± SD)	34.7 ± 11.2	34.4 ± 11.9	0.916
Female (n, %)	25 (43.9)	26 (45.6)	0.864
Initial GCS score (n, %)			0.838
15	25 (43.9)	27 (47.4)	
14	21 (36.8)	21 (36.8)	
13	11 (19.3)	9 (15.8)	
Cause of traumatic brain injury (n, %)			0.782
Motor vehicle accident	34 (59.6)	33 (57.9)	
Fall from height	12 (21.1)	12 (21.1)	
Direct hit on the head	11 (19.3)	12 (21.1)	
Mechanism of traumatic brain injury (n, %)			0.705
Whiplash	7 (12.3)	9 (15.8)	
Head being struck	11 (19.3)	12 (21.1)	
Head striking an object	39 (68.4)	36 (63.2)	
Type of traumatic intracranial hemorrhage (n, %)			-
Subarachnoid	39 (68.4)	-	
Contusional	2 (3.5)	-	
Epidural	17 (29.8)	-	
Subdural	28 (49.1)	-	
Intraventricular	4 (7.0)	-	
Loss of consciousness (n, %)	39 (68.4)	23 (42.6)	0.046
Vomiting (n, %)	19 (33.3)	13 (22.8)	0.194
Tinnitus (n, %)	4 (7.0)	7 (12.3)	0.341
Visual symptoms (n, %)	5 (8.8)	3 (5.3)	0.436
Confusion (n, %)	13 (22.8)	9 (15.8)	0.208
Post-traumatic amnesia (n, %)	15 (26.3)	10 (17.5)	0.145
Post-traumatic seizure (n, %)	6 (10.5)	1 (1.8)	0.005
Previously diagnosed headache (n, %)	9 (15.8)	14 (24.6)	0.172
Antiplatelet agent (n, %)	4 (7.0)	5 (8.8)	0.734
Anticoagulant (n, %)	1 (1.8)	0 (0)	0.681
Initial headache intensity (n, %)			0.079
Mild (NRS 0–3)	14 (24.6)	19 (33.3)	
Moderate-to-Severe (NRS 4–10)	43 (75.4)	38 (66.7)	
PTH at 12-month follow-up (n, %)			0.012
Mild (NRS 0–3)	40 (70.2)	49 (86.0)	
Moderate-to-Severe (NRS 4–10)	17 (29.8)	8 (14.0)	

*Abbreviations*: *GCS* Glasgow Coma Scale, *mTIH* minimal traumatic intracranial hemorrhage, *NRS* numeric rating scale, *PTH* post-traumatic headache, *SD* standard deviation

**Fig. 2:**
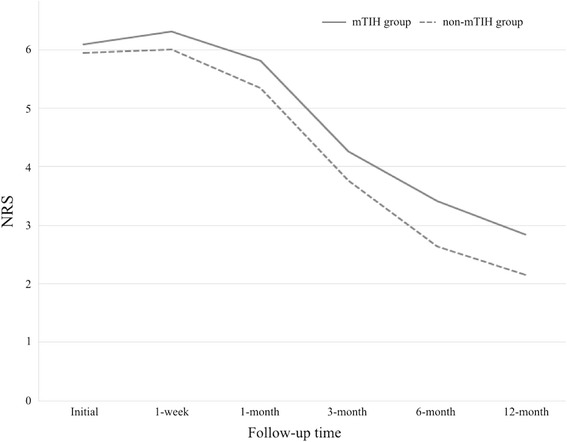
Mean numeric rating scale (NRS) scores of both groups. The difference in NRS scores between mTIH groups and non-mTIH group was statistically significant (95% confidence interval, 0.11 to 1.14; *p* < 0.05, ANCOVA)

More patients experienced LOC after trauma in mTIH group (68.4%) than in non-mTIH group (42.6%, *p* = 0.046). Six (10.5%) of the 57 patients in mTIH group and one (1.8%) of the 57 patients in non-mTIH group experienced post-traumatic seizure, which was a significant difference (*p* = 0.005). Among the six patients with post-traumatic seizure in mTIH group, two patients had contusional hemorrhage and subarachnoid hemorrhage together in the frontal lobe, one had subdural hemorrhage and subarachnoid hemorrhage together in the fronto-temporal lobe, two had subdural hemorrhage in the fronto-parietal lobe, and one had epidural hemorrhage in the parietal lobe. An initial moderate-to-severe headache occurred in 71.1% of patients overall (75.4% in mTIH group and 66.7% in non-mTIH group, *p* = 0.079). At the 12-month follow-up, moderate-to-severe PTH occurred in 21.9% of all patients. Seventeen (29.8%) of the 57 patients in mTIH group had moderate-to-severe PTH, while eight (14.0%) of the 57 patients in non-mTIH group had moderate-to-severe PTH (*p* = 0.012). When the two groups were assessed throughout the 12-month follow-up period, the difference in NRS scores was statistically significant (95% confidence interval, 0.11 to 1.14; *p* < 0.05, ANCOVA), which was consistent over time (Fig. 2).

Multiple logistic regression analysis showed that post-traumatic seizure (odds ratio, 1.520; 95% CI, 1.128–6.785; *p* = 0.047) and mTIH (odds ratio, 2.194; 95% CI, 1.285–8.475; *p* = 0.039) were independently associated with moderate-to-severe PTH (NRS ≥ 4) at the 12-month follow-up (Table 2). Initial headache intensity had a *p*-value of 0.044 in simple logistic regression analysis, but was not statistically significant after adjustment (*p* = 0.094).

**Table 2:** Risk factors for moderate-to-severe headache at the 12-month follow-up

	Simple logistic regression		Multiple logistic regression	
	OR (95% CI)	*p* value	OR (95% CI)	*p* value
Age in years				
<35	1			
≥35	1.407 (0.529–3.106)	0.748		
Gender				
Female	1			
Male	0.912 (0.478–2.106)	0.649		
Initial GCS				
15	1			
<15	1.794 (0.593–3.377)	0.573		
Cause of traumatic brain injury				
Motor vehicle accident	1			
Fall from height	2.975 (0.636–4.647)	0.443		
Direct hit on the head	0.846 (0.743–3.226)	0.384		
Mechanism of traumatic brain injury				
Whiplash	1			
Head being struck	1.839 (0.475–7.552)	0.751		
Head striking an object	2.883 (0.748–3.424)	0.395		
Loss of consciousness				
No	1		1	
Yes	2.174 (0.944–3.806)	0.078	2.311 (0.839–4.394)	0.162
Vomiting				
No	1			
Yes	2.082 (0.624–4.771)	0.736		
Tinnitus				
No	1			
Yes	0.859 (0.344–1.176)	0.611		
Visual symptoms				
No	1			
Yes	1.075 (0.525–3.239)	0.522		
Confusion				
No	1			
Yes	1.742 (0.438–2.262)	0.704		
Post-traumatic amnesia				
No	1			
Yes	2.242 (0.748–5.680)	0.663		
Post-traumatic seizure				
No	1		1	
Yes	1.779 (1.205–4.430)	0.035	1.520 (1.128–6.785)	0.047
Previously diagnosed headache				
No	1		1	
Yes	1.835 (0.806–4.704)	0.082	1.503 (0.724–5.960)	0.179
Initial headache intensity				
Mild (NRS 0–3)	1		1	
Moderate-to-Severe (NRS 4–10)	2.927 (1.348–4.612)	0.044	2.154 (0.925–6.319)	0.094
mTIH				
No	1		1	
Yes	2.883 (1.038–6.124)	0.021	2.194 (1.285–8.475)	0.039

*Abbreviations*: *CI* confidence interval, *GCS* Glasgow Coma Scale, *mTIH* minimal traumatic intracranial hemorrhage, *NRS* numeric rating scale, *OR* odds ratio

## Discussion

Patients with mTIH after TBI were more likely to experience LOC and post-traumatic seizure than were patients without mTIH. Throughout the 12-month follow-up, NRS scores in patients with mTIH were significantly higher than in those without mTIH. The overall occurrence rate of moderate-to-severe PTH was 21.9% at the 12-month follow-up. Moderate-to-severe PTH might be expected in patients with post-traumatic seizure and mTIH after TBI.

The reported prevalence of PTH due to TBI ranges between 20 and 89% [2, 10–12]. Two recent studies examined the rate of headache longitudinally among a large cohort during the first year after TBI [13, 14]. The incidence at baseline was 44–54%, and the cumulative incidence of headache over 1 year was 71–91%. More than one-third of the subjects reported persistent headache throughout the follow-up period. In the present study, the prevalence of PTH (mild, moderate, and severe PTH) was 57.9% at the 12-month follow-up, which seemed to be higher than the previously reported prevalence. We focused on moderate-to-severe PTH, which might have more clinical relevance, because mild PTH (NRS 1 to 3) was described as interfering little with activities of daily living, while moderate-to-severe PTH (NRS 4 to 10) interfered with or prevented activities of daily living.

Pathophysiology and characteristics of PTH in patients with mTIH after TBI are not well understood. Consequently, the best treatment options for PTH remain unclear. Although PTH affects patient quality of life, patients with mTIH after TBI tends to be neglected in the real world because the clinical focus is to achieve a good outcome (to minimize morbidity and mortality) in patients with severe intracranial findings. However, we felt that it was worthwhile to study the incidence and risk factors of PTH in patients with mTIH after TBI. We identified two risk factors, mTIH and post-traumatic seizure, that were related to moderate-to-severe PTH at the 12-month follow-up in patients with mTIH after TBI. These data may allow providers to educate their patients about what they may expect with regards to PTH after mTIH.

Presence of mTIH after TBI suggests a greater impact force on the brain than absence of mTIH after TBI. This may explain why mTIH group had more patients who experienced LOC, post-traumatic seizure, and PTH than non-mTIH group in the present study. Post-traumatic seizure is more likely to occur in more severe injuries. Post-traumatic seizure that occurs immediately after TBI is thought to be a direct reaction to the injury. This happens because the force from the injury stimulates brain tissue that has a low threshold for seizures when stimulated [15, 16]. Early post-traumatic seizure can be caused by factors such as intracranial hemorrhage or cerebral contusion [15, 17]. Since we have not analyzed the relationship between post-traumatic seizure and types of mTIH, it was not clear if post-traumatic seizure was related to any type of mTIH in the present study. It is known that PTH after TBI can be caused by axonal shear, resulting in incomplete neuronal transmission, or by induction of trigeminal nociception, which subsequently results in cortical spreading depression [18]. The impact force of the trauma that produced the mTIH might provoke PTH by inducing incomplete neuronal transmission or cortical spreading depression. In the same context, 54.4% (62/114) of LOC (68.4% in mTIH group and 42.6% in non-mTIH group) in the present study, that seemed a bit high for patients with mild TBI, can be understood. The initial impact to the head could be more severe in patients of the present study than those with mild TBI in general because a half of our patients had visible abnormalities on structural neuroimaging. Beetar et al. have previously reported that 62.9% (127/202) of their patients with mild TBI had LOC [19].

Based on our findings, brain CTs to detect mTIH in patients with TBI may be important because mTIH was associated with moderate-to-severe PTH throughout the 12-month follow-up period. There is significant disagreement about the indications for brain CT scan in such patients after TBI because of the cost, time, and probable complications of radiation [20]. Symptoms such as headache, vomiting, LOC, amnesia, post-traumatic seizure, skull fracture, contusion, raccoon sign, alcohol intoxication, coagulopathy, and age more than 60 years have been proposed as risk indicators of abnormalities in brain CT scans of patients with TBI; if any patient has these indicators following TBI, the patient should be considered to be at high-risk for abnormal CT findings [21]. However, no consensus has been achieved in the indications for brain CT scan. Nevertheless, headache by itself, as an initial symptom, can be used as a guide to predict the probability of an abnormal brain CT scan related to TBI [22, 23]. In our institution, we performed brain CT scans of as many patients with newly developed headache after head trauma as possible, but not in all patients. This is one of the limitations of our study and is described in detail below. We could not include some patients with mTIH because they did not have radiographic images. Patients with neurologically asymptomatic mTIH after TBI require close observation not only because of aggravation of hematoma and edema in the acute period, but because of PTH during the follow-up period. Risk factors of PTH should not be neglected, and appropriate medications should be prescribed for patients with mTIH who develop PTH after TBI.

There are several limitations to this study. First, this was a retrospective study with a small number of patients; therefore, there is potential for selection bias even though we performed propensity score matching. We did not have a database of patients who did not undergo CT scan after head injury. Because we required radiographic evidence of traumatic intracranial hemorrhage, we could not include those patients who did not have radiographic images. These patients may have had mTIH initially or PTH afterward, which could have altered our findings. Second, we also did not compare headache characteristics, such as type, onset, duration, or frequency, between groups. The most frequent types of PTH are migraine, tension-type headache, and cervicogenic headache [13]. Our results may have been different if we had evaluated PTH characteristics. However, our focus was headache intensity as assessed by a subjective score (NRS score), because we were interested in PTH in general and its potential effects on activities of daily living. In addition, we included patients who did not have neurological disability with GCS ≥ 13 because disability could affect PTH. We, also, excluded patients with spinal cord injury because some patients with TBI could have headaches secondary to spinal cord injury. Third, we did not obtain sufficient information about headache medications. Although medications may be an important factor affecting headache improvement, it was very difficult to analyze when or how medications were used, preventing such an analysis. However, we attempted to evaluate the correlation between previously physician-diagnosed headache treated by medication and moderate-to-severe PTH at the 12-month follow-up. Fourth, we could not have defined the type of post-traumatic seizures (generalized tonic-clonic, partial, or partial complex seizures) nor have had the results of electroencephalogram in all patients who experienced post-traumatic seizure since our database only has clinical signs of post-traumatic seizure. Finally, we did not evaluate symptoms related to cognitive, behavioral, or emotional dysfunction. We only evaluated and focused on a single physical symptom, PTH. Cognitive symptoms may result from dysfunctions of attention, concentration, memory, processing speed, judgment, and executive functioning. Behavioral or emotional symptoms may include depression, anxiety, agitation, irritability, and aggression. It is believed that those cognitive, behavioral, and emotional symptoms resulting from mTIH after TBI are inter-related; alleviation of one symptom often leads to improvement in others [24]. Consequently, aggressive treatment of any comorbid psychiatric illness may help to improve PTH, and vice versa. Thus, these symptoms may be risk factors of PTH after TBI and have important clinical implications. Future research is needed to evaluate the role of these kinds of symptoms in the development of PTH.

## Conclusions

Moderate-to-severe PTH might occur after TBI in patients with mTIH and post-traumatic seizure. PTH appears to occur more frequently in patients with mTIH than in those without mTIH. In patients with mTIH, letting them be aware of what they can expect with regards to PTH after mTIH might be necessary.

## Abbreviations

ASD: Absolute standardized differences
CI: Confidence interval
CT: Computed tomography
GCS: Glasgow Coma Scale
LOC: Loss of consciousness
MRI: Magnetic resonance image
mRS: Modified Rankin Scale
mTIH: Minimal traumatic intracranial hemorrhage
NRS: Numeric rating scale
PTH: Post-traumatic headache
TBI: Traumatic brain injury
